# Hydrophobin‐Coated Echogenic Microbubbles for Molecular Targeting of Tumor Cells

**DOI:** 10.1002/advs.202401526

**Published:** 2025-05-19

**Authors:** Hedar H. Al‐Terke, Grégory Beaune, Muhammad Junaid, Jani Seitsonen, Arja Paananen, Pierangelo Metrangolo, Jaakko V. I. Timonen, Jussi Joensuu, Robin H. A. Ras

**Affiliations:** ^1^ Department of Applied Physics Aalto University School of Science Espoo FI‐02150 Finland; ^2^ Center of Excellence in Life‐Inspired Hybrid Materials (LIBER) Espoo FI‐02150 Finland; ^3^ VTT Technical Research Centre of Finland Ltd. P.O. Box 1000, VTT Espoo FI‐02044 Finland; ^4^ Department of Chemistry Materials and Chemical Engineering “Giulio Natta” Politecnico di Milano Milano IT‐20131 Italy

**Keywords:** hydrophobins, molecular imaging, targeted microbubbles, tumor cells, ultrasound contrast agents

## Abstract

Cancer is a leading cause of death globally. Early detection of tumors can be an effective way to reduce mortality, however, traditional cancer diagnostic methods are based on the detection of organ dysfunction, which generally occurs at advanced stages of the disease. The first signs of disease initiation appear much earlier as molecular abnormalities yet are challenging to detect. An advanced design of microbubbles is reported that allows molecular targeting for subsequent binding to tumor cells. The microbubbles consist of perfluorocarbon gas encapsulated in a functional and stable shell made of a surface‐active protein (hydrophobin HFBI from *Trichoderma reesei*) and corresponding fusion protein (HFBI‐domZ) onto which antibodies can be easily grafted. The functionality of the bubbles is investigated, among others, using cryogenic transmission electron microscopy (cryo‐TEM), confocal microscopy, and in vitro experiments. The results show the potential of the microbubbles as a probe to be used as a targeting contrast agent in ultrasound molecular imaging (MI) for cancer diagnostics.

## Graphical abstract

Echogenic microbubbles based on hydrophobin and corresponding fusion protein (HFBI‐domZ) are developed as contrast agents for cancer diagnostics. Hydrophobin provides high stabilization of encapsulated perfluoro‐n‐butane, and fusion proteins allow further versatile functionalization with antibodies. In‐vitro experiments indicate that these microbubbles can target tumor cells and be observed with ultrasound imaging.

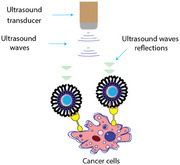

## 1 Introduction

Medical imaging is an essential part of routine health care. It is used as an important diagnostic tool for the detection and control of diseases. Modern medical imaging comprises techniques such as X‐ray, positron emission tomography (PET), magnetic resonance imaging (MRI), computed tomography (CT), and ultrasound imaging.^[^ ^1^, ^2^, ^3^ ^]^ These modalities share a common philosophy: they are designed to detect abnormalities in the structure or physiology of organs. However, these dysfunctions often indicate an advanced stage of disease, while the initial stages usually remain invisible. Consequently, current medical imaging modalities fail to capture the critical early period of disease development, which is crucial for timely disease control and early intervention.

Biomedical research focuses on modalities enabling the visualization, characteri‐zation, and quantification of biological behavior at the cellular and subcellular scales, called molecular imaging. It describes molecular and cellular interactions and disease mechanisms in a living body.^[^ ^4^, ^5^, ^6^ ^]^ Typically, MI is applied to establish the basis of a disease by studying molecular abnormalities in cells. It combines one or two conventional medical imaging modalities with a predesigned advanced probe. These probes consist of two main components that are linked together. The first component is the signaling source, while the second is the recognition moiety, which enables the probe to specifically bind to the target.^[^ ^7^ ^]^ For instance, the signaling parts are magnetic nanoparticles for MRI, bioluminescent and fluorescent molecules for optical microscopy, or nano‐microbubbles for ultrasound imaging.^[^ ^5^, ^8^, ^9^, ^10^ ^]^ On the other hand, antibodies, viruses, proteins, and small molecules can serve as moiety parts (which can attach to the antigens), which recognize and attach to the target on the surface of abnormal cells.^[^ ^7^ ^]^ In addition to these crucial structural parts, the MI probe requires several key features, including high binding affinity and specificity to the target, high sensitivity, excellent imaging contrast, as well as cost‐effective production and feasibility.^[^ ^5^ ^]^ The limitations of high‐quality MI techniques such as PET/CT and PET/MRI are that they are typically expensive and are based on radioactive materials.^[^ ^2^ ^]^ In contrast to these radioactive techniques, the ultrasound imaging technique is safer and more cost‐effective.

Ultrasound imaging is the second most commonly used method after X‐ray in medical imaging.^[^ ^11^ ^]^ It exposes the body to high‐frequency sound waves reflected at different interfaces. Subsequently, an image is generated by calculating the time elapsed for the ultrasound wave to leave and return to the transducer and its amplitude.^[^ ^5^, ^12^, ^13^, ^14^ ^]^ Generally, the signal part of the ultrasound molecular imaging probe consists of substances that can reflect ultrasound waves stronger than surrounding tissues called contrast agents, such as nano and microbubbles or liposomes.^[^ ^15^, ^16^ ^]^ Microbubbles are used as contrast agents for non‐targeted ultrasound imaging, such as in the diagnosis of heart, liver, and kidney diseases.^[^ ^17^ ^]^ Microbubbles can be injected intravenously to enhance contrast due to their mechanical properties, which cause a stronger reflection of the incident ultrasound wave than the wave reflected from the tissue. They typically consist of a gaseous core that is non‐polar to improve stability in aqueous media and an interfacial shell typically made of phospholipids, proteins, or polymers to create a viscoelastic layer that stabilizes the gaseous core and reflects the ultrasound waves.^[^ ^18^, ^19^ ^]^ The gas of the bubbles is normally secreted from the body by exhalation via the lungs. The shell materials are metabolized by the body, eliminated by the liver, or filtered by the kidneys.^[^ ^20^ ^]^ Commercially available non‐targeted microbubbles like Definity (Lantheus, USA), Lumason (Bracco, Italy), Optison (GE HealthCare, USA), and Sonazoid (Daiichi–Sankyo, Japan) are generally made of phospholipids or albumin protein. We focus here on microbubbles, as nanobubbles and liposomes are still in the research stage due to echogenicity (reflection of ultrasound waves) and stability challenges.^[^ ^21^, ^22^ ^]^

Bubbles can be non‐targeting, meaning they have no specific affinity to attach to any target, or they can be functionalized with a particular moiety that can recognize specific cells and adhere to a target tissue. Bubbles allow the visualization of blood and blood flow within an organ by differentiating between normal and abnormal conditions. Therefore, the quality of the ultrasound image is highly related to the echogenicity of the contrast agent.

Phospholipid‐based microbubbles have excellent echogenicity due to the mechanical properties of their shell.^[^ ^23^ ^]^ However, they present a very short lifetime (several minutes) under the relatively high pressure inside the blood vessels (≈130 mbar), which limits their practical use.^[^ ^19^, ^24^ ^]^ Polymer‐based microbubbles have a longer lifetime yet a weak echogenic signal due to the rigidity of their shell.^[^ ^25^ ^]^ Cen et al. recently reported improved signal and stability of a copolypeptide‐based contrast agent.^[^ ^26^ ^]^ Bubbles based on surface‐active proteins have viscoelastic properties between phospholipids and polymers, which make them a better alternative for ultrasound contrast agent applications that require high stability for periods of time long enough to complete the diagnostic process (between 5 and 15 min, depending on the nature of the contrast agent).^[^ ^27^, ^28^ ^]^

Our previous work investigated the mechanical properties of a unique surface‐active protein HFBI, which belongs to the family of highly surface‐active fungal proteins (Class II hydrophobins).^[^ ^29^ ^]^ Hydrophobins are relatively small, Janus‐like proteins produced by filamentous fungi.^[^ ^30^ ^]^ Due to their amphiphilic nature, they tend to self‐organize into a monolayer at various interfaces. They have eight highly conserved cysteine residues forming four disulfide bonds (S─S),^[^ ^31^, ^32^ ^]^ which gives them high stability at interfaces and rigid biological behavior similar to nanoparticles.^[^ ^33^ ^]^ The study showed that the mechanical properties of gas bubbles encapsulated with a monolayer of HFBI are suitable for ultrasound applications. In addition to our previous work, there are several reports about the HFBII protein that is also derived from *Trichoderma reesei* fungi. HFBII has been used as a bubble stabilizer in these studies.^[^ ^34^, ^35^, ^36^, ^37^ ^]^ It has been shown that HFBI and HFBII show different behaviors at the air‐water interface. Specifically, HFBI bubbles might be more stable and HFBI could interact more strongly at the bubble interface than HFBII.^[^ ^38^ ^]^ It is hypothesized that these differences between the two proteins are related to divergence in the size and shape of their hydrophobic moieties.

Hydrophobins can be fused with other functional elements such as proteins, enzymes, or other functional ligands to get a multifunctional complex.^[^ ^39^, ^40^, ^41^, ^42^ ^]^ For instance, the HFBI‐protein A fusion protein combines the HFBI protein and protein A from *Staphylococcus aureus*.^[^ ^43^, ^44^ ^]^ This fusion protein is bifunctional because it can create a monolayer at the liquid‐gas interface, which allows versatile binding and release of Immunoglobulin G (IgG) antibody molecules.^[^ ^40^ ^]^ Protein A consists of five domains, each capable of binding an IgG molecule by the formation of numerous noncovalent bonds between the amino acids of the binding sites, providing a straightforward way to functionalize the bubbles by targeting IgG antibodies.^[^ ^45^, ^46^ ^]^

Cancer cells have specific biomarkers (antigens) such as proteins, peptides, lipids, polysaccharides, or nucleic acids, that can trigger the immune system and bind to T‐cell receptors or specific antibodies. Antibodies can recognize antigens via specific surface features called epitopes, which can be identified by specific antibody paratopes.

The current study focuses on the complete design of an ultrasound molecular imaging probe based on surface‐active hydrophobin proteins (HFBI and its fusion protein HFBI‐domZ). HFBI‐domZ fusion protein is a protein that consists of one HFBI molecule covalently connected with one functional domain from protein A

A mixture of HFBI and its fusion protein HFBI‐domZ, is used to form echogenic microbubbles. This mixture of molecules self‐assembles into a bifunctional layer at the interface between perfluoro‐*n*‐butane gas (C_4_F_10_) and phosphate‐buffered saline (PBS), in which the microbubbles are dispersed. The bubbles containing domZ moieties are then functionalized with monoclonal antibodies that can bind to specific antigens on the surface of targeted cells. This allowed us to decorate our bubbles with the desired antibody for known target epitopes. The bubbles presented here are echogenic and programmable molecular targeting agents.

## 2 Results and Discussion

### 2.1 Ultrasound Molecular Imaging Probe Architecture

**Figure 1:**
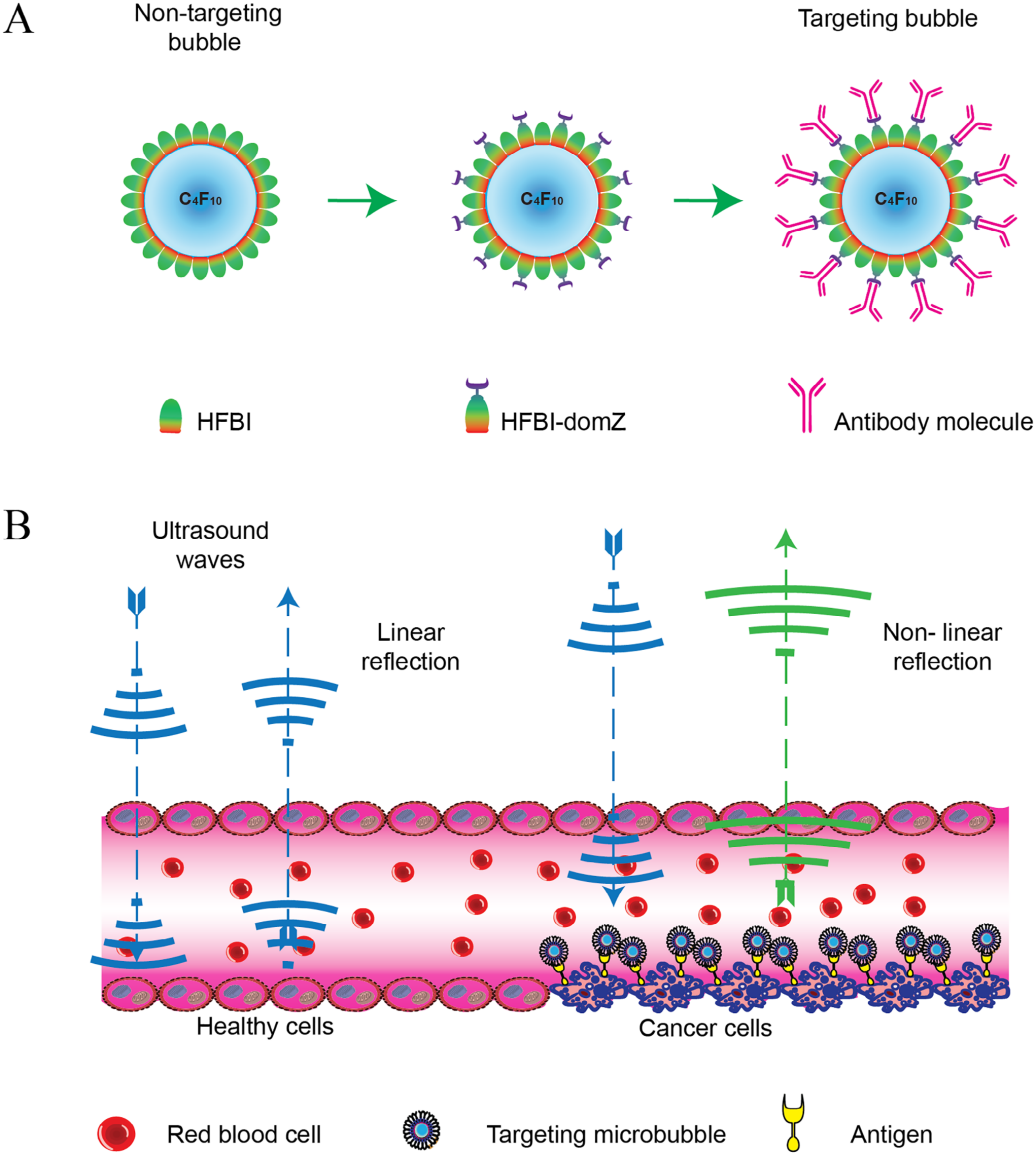
Schematic illustration of HFBI‐based gas bubbles as an ultrasound contrast agent. (A) Structure of non‐targeting and targeting echogenic microbubbles. (B) Ultrasound imaging contrast is provided by the echogenic microbubbles decorated with specific antibody molecules that attach to the antigen on the surface of cancer cells.

C_4_F_10_ gas bubbles encapsulated with native‐type HFBI can serve as a non‐targeting contrast agent (**Figure** **1**A). The use of a mixture of native‐type HFBI and HFBI‐domZ fusion protein creates functional bubbles that can be decorated with a specific monoclonal antibody to target a particular antigen on the surface of cancer cells (Figure 1B).

### 2.2 Surface Activity and Mechanical Properties

**Figure 2:**
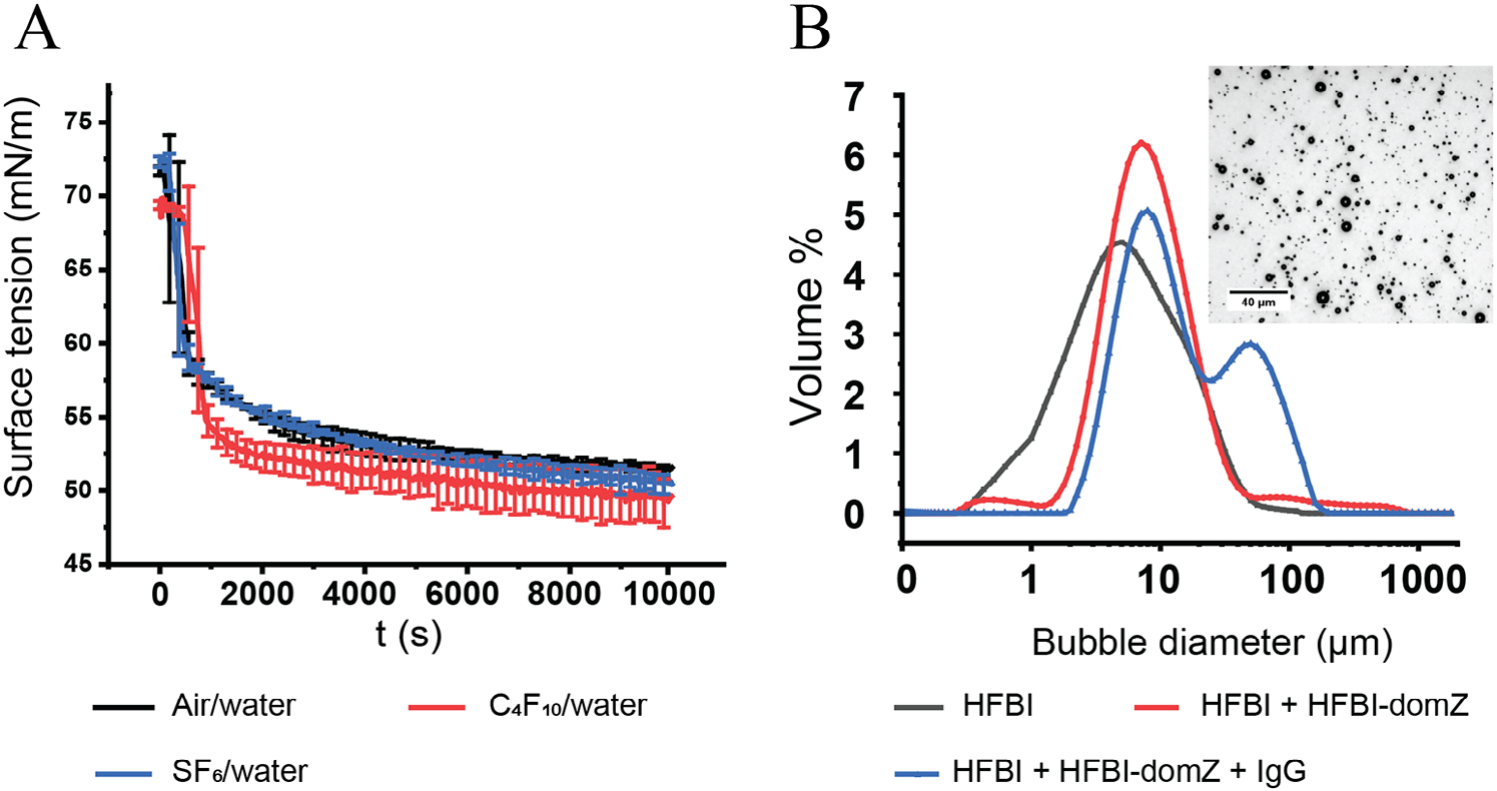
(A) Surface activity of HFBI‐domZ and (B) bubble size distribution measured using Mastersizer particle analyzer. The inset of (B) is an optical microscopy image of bubble suspension.

Hydrophobins and their fusion proteins, such as HFBI‐domZ, are highly surface active due to their amphiphilic nature. Therefore, they adsorb at gas‐liquid interfaces as a viscoelastic monolayer.^[^ ^36^ ^]^ The adsorption rate and the mechanical properties of the viscoelastic layer play an essential role in bubble generation and their functional properties. The adsorption rate is affected by the bulk concentration and the surface activity of the molecules. The surface activity of the HFBI‐domZ fusion protein was investigated using three gases: air, C_4_F_10_, and sulfur hexafluoride (SF_6_). Air is used as a reference, while C_4_F_10_ and SF_6_ are gases of choice for the aimed applications because they are highly non‐polar and heavier than air in aqueous solutions.^[^ ^17^ ^]^ The adsorption of the protein molecules to the interface significantly lowers the surface tension, as demonstrated by the sharp slope of the surface tension curve, where the values decrease by more than 20 mN m^−1^ (**Figure** **2**A). In addition, the curves almost reach the steady state within 35 min, confirming the high affinity of the HFBI‐domZ molecules with gas‐liquid interfaces.

The mechanical properties of the protein layers at the interface were measured using the axisymmetric bubble shape analysis method. **Table** **1** shows the storage (*E′*) and loss (*E″*) moduli of the layers. The storage moduli were almost identical for the layers formed at the air‐water and SF_6_‐water interfaces. However, the layer at the C_4_F_10_‐water interface has a slightly higher storage modulus and smaller loss modulus, meaning that the layer dissipates more energy in compression and induces less resistance in expansion. In other words, the layer is more viscoelastic compared to the layers adsorbed at air‐water and SF_6_‐water interfaces, which makes it the best choice for non‐linear ultrasound wave reflection applications. In addition to the mechanical properties of the adsorbed layer, perfluorocarbon gases play a unique role in bubble stability (half‐life time). They have an extremely low water solubility (0.021 mol m^−3^ for C_4_F_10_), which means that the gas molecules remain inside the bubbles, creating a pressure that balances the surface tension and the blood pressure.^[^ ^19^ ^]^ For these reasons, we focus on the microbubbles produced with C_4_F_10_ in the rest of this work.

**Table 1:** Storage and loss moduli of HFBI‐domZ fusion protein layer adsorbed at air, SF_6,_ and C_4_F_10_ gases.

Interface	*E′* (mN/m)	*E″* (mN/m)
**Air / water**	126 ± 18	18 ± 8
**SF_6_ / water**	124 ± 7	16 ± 2
**C_4_F_10_ / water**	86 ± 6	21 ± 6

### 2.3 Size Distribution and Zeta Potential

The typical size of ultrasound contrast agent bubbles ranges from submicron to 8 microns in diameter. They are large enough (on the order of red blood cells) to ensure high echogenicity and small enough to travel smoothly in the blood circulatory system.^[^ ^17^ ^]^ We determine the size distribution of the bubbles, verify the introduction of HFBI‐domZ in the monolayer, and the functionalization of the bubbles with IgG, by performing dynamic light scattering and zeta potential (ZP) measurements. We characterize bubbles with three different formulations: native‐type hydrophobin HFBI, 20% HFBI‐domZ fusion protein +80% HFBI, and 20% HFBI‐domZ + 80% HFBI + IgG.

The size distribution of the bubbles shows that the peaks of the number percentage of the two first formulations are very similar with values of 0.7 and 0.5 µm. We also observe that after the addition of HFBI‐domZ, we have a less intense second population of bubbles with a peak at 2.5 µm. Finally, for the formulation including IgG, we observe a peak of a population with a larger diameter of 3.2 µm (Figure 2B). The size distribution of the bubbles has also been plotted in number (Figure S1, Supporting Information) and it also shows an increase of the peaks from 0.7 µm for HFBI stabilized bubbles to 3.2 µm for IgG functionalized bubbles.

The bubbles coated with HFBI have the smallest size and lowest ZP (−66 ± 6 mV). These negatively charged bubbles can easily create a repulsion force between each other and prevent coagulation. In the case of HFBI + HFBI‐domZ, the average bubble size increases, and the ZP is slightly less negative (−54 ± 6 mV). These changes can be interpreted as indirect evidence of the adsorption of HFBI‐domZ molecules at the interface. This result is confirmed using AFM (Figure S2, Supporting Information). The AFM images show two types of areas at the interface, which can be interpreted as two types of proteins based to their size differences. On the other hand, the significant change in the ZP of the bubbles decorated with IgG molecules (15 ± 2 mV) is an indirect proof of the attachment between IgG molecules and the surface of the bubbles. Indeed, the ZP of IgG is ≈15 mV in pH ≈5.5, which is the pH value of Milli‐Q water.^[^ ^47^ ^]^ This slightly positive value can cause some bubbles to aggregate, which explains the second peak in the size distribution curve (Figure 2B, blue curve). From the size distributions we obtained, we calculated the percentage of microbubbles (in volume and in number) that are suitable for different medical applications (Table S1, Supporting Information). It indicates that their size is suitable for ultrasound imaging, as expected, but interestingly, they could also be of interest for other applications related to blood perfusion, inflammation, and tumor angiogenesis.^[^ ^48^ ^]^

### 2.4 Stability of Bubbles Under High Pressure

As mentioned earlier, bubbles must withstand relatively high blood pressure ≈130 mbar. They must also be able to stretch and compress to reflect non‐linear ultrasound echoes. Since normal blood pressure is ≈130 mbar, bubbles must survive at least such pressures. HFBI forms a rigid monolayer at gas interfaces. Mixing HFBI‐domZ with HFBI makes the layer more elastic (Figure S3, Supporting Information), which could affect the stability of the bubbles under high pressure. For percentages up to 30% HFBI‐domZ, HFBI dominates the interface and leads to a misfitting of the Laplace equation due to the rigid behavior of HFBI. On the other hand, for higher HFBI‐domZ percentages, such as 40 and 50%, the layer behaves like an elastic layer. Therefore, we focused in this study on bubbles that have protein shells made from a solution of 20% HFBI‐domZ + 80% HFBI in PBS buffer.

**Figure 3:**
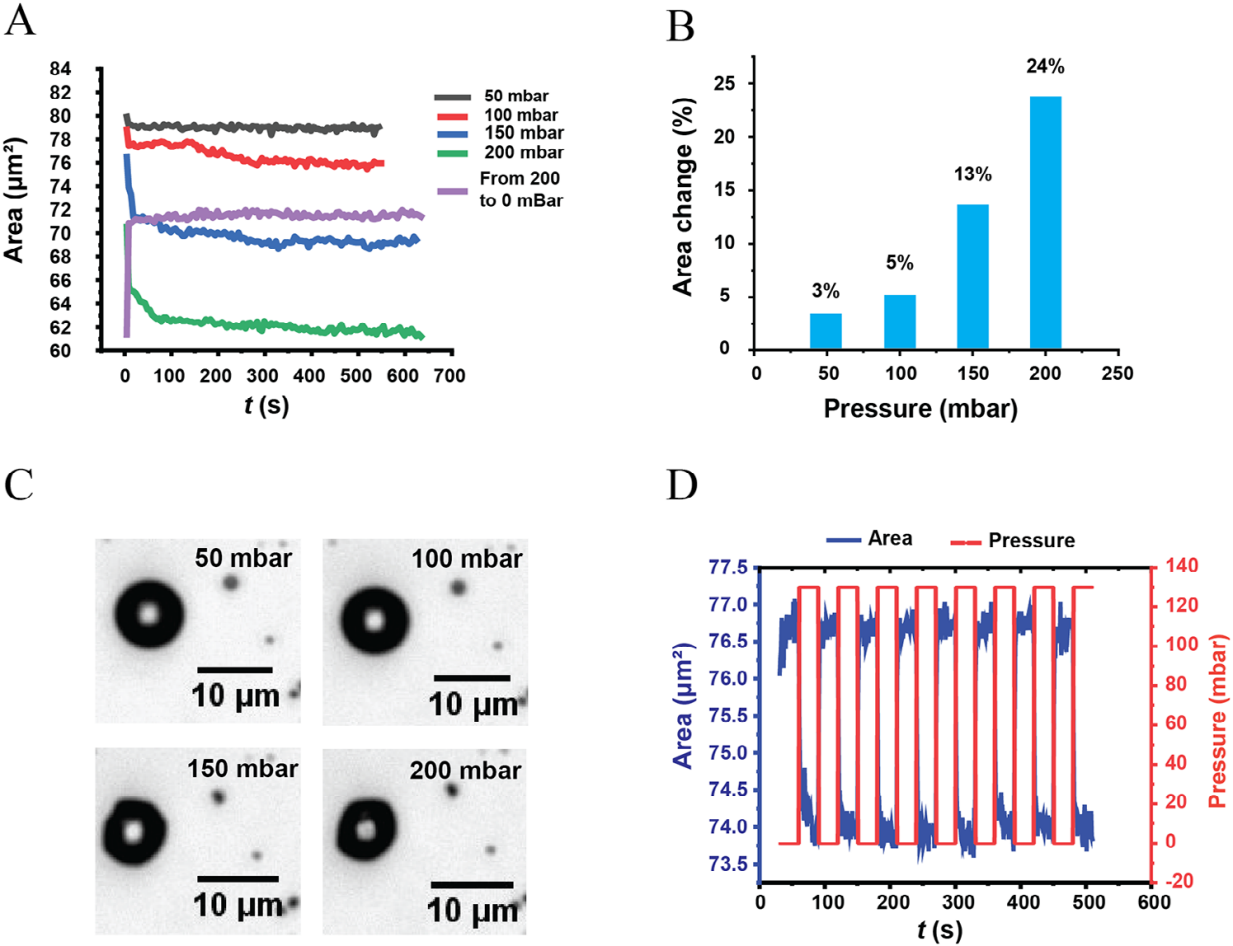
Stability of bubbles under pushing pressure. (A) The changes in the cross‐section area of a bubble as the pressure increases in 50 mbar increments up to 200 mbar, followed by a release of pressure. (B) The percentage change in the cross‐section area of the same bubble for the different pressures. (C) Optical microscopy images of a bubble at four different pressures. (D) The response of a bubble to repeated radical stress changes.

A homemade setup was used to measure the bubble stability. An external pushing pressure was applied to bubbles located inside a capillary, and the evolution of their cross‐section area was monitored using an inverted optical microscope. The pressure was increased from 0 to 200 mbar above atmospheric pressure in 50 mbar increments, and each step was maintained for 10 min to reach a steady state (**Figure** **3**A). The cross‐section area decreases with each step by 3, 5, 13, and 24%, respectively (Figure 3B,C). This means that even though the pressure was much higher than normal blood pressure, bubbles shrank by less than 25% of their original size. In addition, when the external pressure returned to the ambient pressure ≈1 bar, the bubble quickly recovered 80% of its original volume. This means that only 20% of C_4_F_10_ diffused to the bulk solution at 200 mbar pressure.

Furthermore, the response of the bubbles to repeated radical pressure change was observed. When the external pressure was periodically alternated between 0 and 130 mbar (Figure 3D), the bubble size responded harmonically, which is crucial for non‐linear ultrasound echoes. In addition to the high pushing pressure, bubbles respond to aspiration pressures, and their cross‐section areas increase when the aspiration pressure increases, confirming the elasticity of the protein layer (Figure S4, Supporting Information).

### 2.5 Bubbles Decoration with Antibody Molecules

The main difference between targeting and non‐targeting microbubbles is their ability to recognize targeted biomarkers on the surface of abnormal cells. Bubbles decorated with a moiety part like polypeptides, proteins, or antibodies can identify the targeted biomarkers and adhere to the cell surface. As shown in the AFM images (Figure S2, Supporting Information), the interface of the bubbles consists of HFBI‐domZ and HFBI proteins. HFBI‐domZ can bind to IgG antibodies by recognizing their FC (fragment crystallizable region) part, making the bubble fully covered with antibody molecules that have free Fab (fragment antigen‐binding region) parts to recognize and attach to the antigen on the surface of the infected cells.

**Figure 4:**
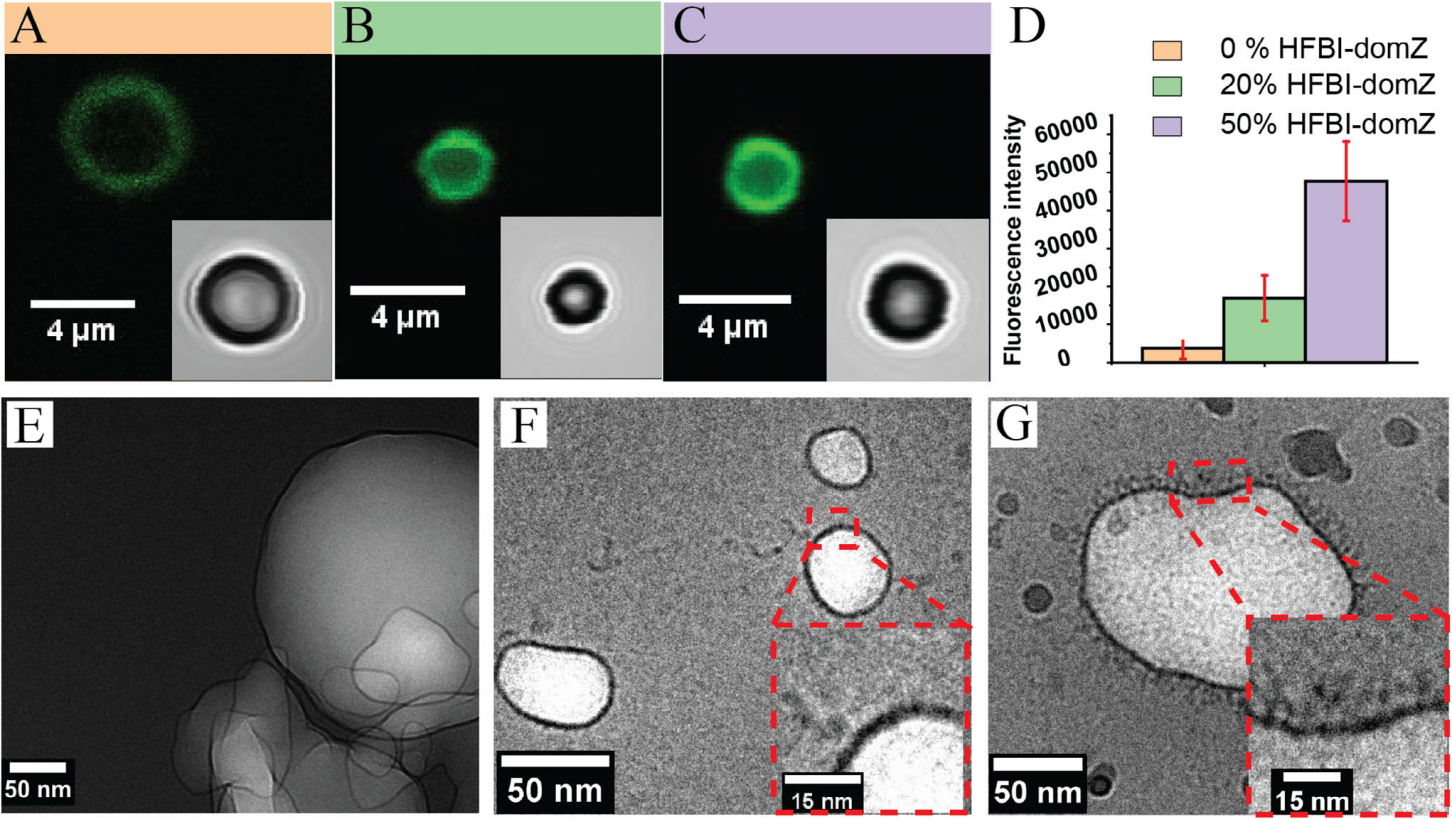
Bubbles decorated with antibody molecules. Confocal microscope images (A–C) and cryo‐TEM images (E–G). Bubbles stabilized with (A) native‐type hydrophobin (HFBI), (B) 20% HFBI‐domZ + 80% HFBI, (C) 50% HFBI‐domZ + 50% HFBI, all 3 after the addition of fluorescent IgG (IgG‐NHS‐Fluorescein). The inset images in A, B, and C are the optical images of the same bubbles in a bright field. (D) The fluorescence intensities of the bubbles with the different compositions. Cryo‐TEM images of (E) bubbles stabilized with HFBI, as well as bubbles stabilized by a mixture of 20% HFBI‐domZ + 80% HFBI (F) before and (G) after decoration with IgG. The insets in F and G are magnified images showing the shells of the bubbles.

Confocal laser scanning microscopy and cryo‐TEM were used to characterize the decoration of the HFBI + HFBI‐domZ‐based microbubbles with IgG antibodies. As explained in the experimental section, three batches of bubbles encapsulating C_4_F_10_ were prepared and investigated. **Figure** **4**A–D show a significant increase in fluorescence intensity with an increasing percentage of HFBI‐domZ. It demonstrates the decoration of the bubbles with IgG and shows the linear correlation between the number of HFBI‐domZ in the bubble structure and the attachment of IgG‐NHS‐Fluorescein molecules. The average fluorescence intensity was ≈3600 (arbitrary unit) when the bubbles were coated with HFBI only. While when the layer contained HFBI‐domZ molecules adsorbed from solutions of 20% HFBI‐domZ + 80% HFBI and 50% HFBI‐domZ + 50% HFBI, the fluorescence intensities increased to 17 000 and 48 000, respectively. The low value of fluorescence intensity when there is no HFBI‐domZ is expected due to the non‐selective adsorption of IgG on the bubble surface. In contrast, the high value is due to the selective attraction between the domZ and the FC part of the IgG.

Cryo‐TEM also confirms the binding between the domZ and the FC part of the IgG. Nanobubbles from three batches of micro‐nanobubbles were observed. The microbubbles could not be observed due to the size limitation of cryo‐TEM. The first batch consists of bubbles with a HFBI shell (Figure 4E). The second batch of bubbles has a shell of proteins adsorbed from a solution of 80% HFBI + 20% HFBI‐domZ (Figure 4F). The third batch of bubbles has a shell as the second batch and is functionalized with IgG (Figure 4G). The shell thickness of the HFBI bubbles was 2.7 ± 0.4 nm, corresponding to the length of one HFBI molecule. The HFBI‐domZ molecules made the shell a bit thicker, with a thickness of 3.3 ± 0.3 nm. Nevertheless, the shells remained smooth in both cases, as shown in Figure 4E,F. On the other hand, Figure 4G confirms the functionalization of HFBI‐domZ with IgG with the observation of a shell with a hairy structure. The hairy region was 9.4 ± 1.2 nm thick, which matches the length of one IgG molecule.

### 2.6 Antigen Recognition and Binding to the Surface of Cancer Cells

**Figure 5:**
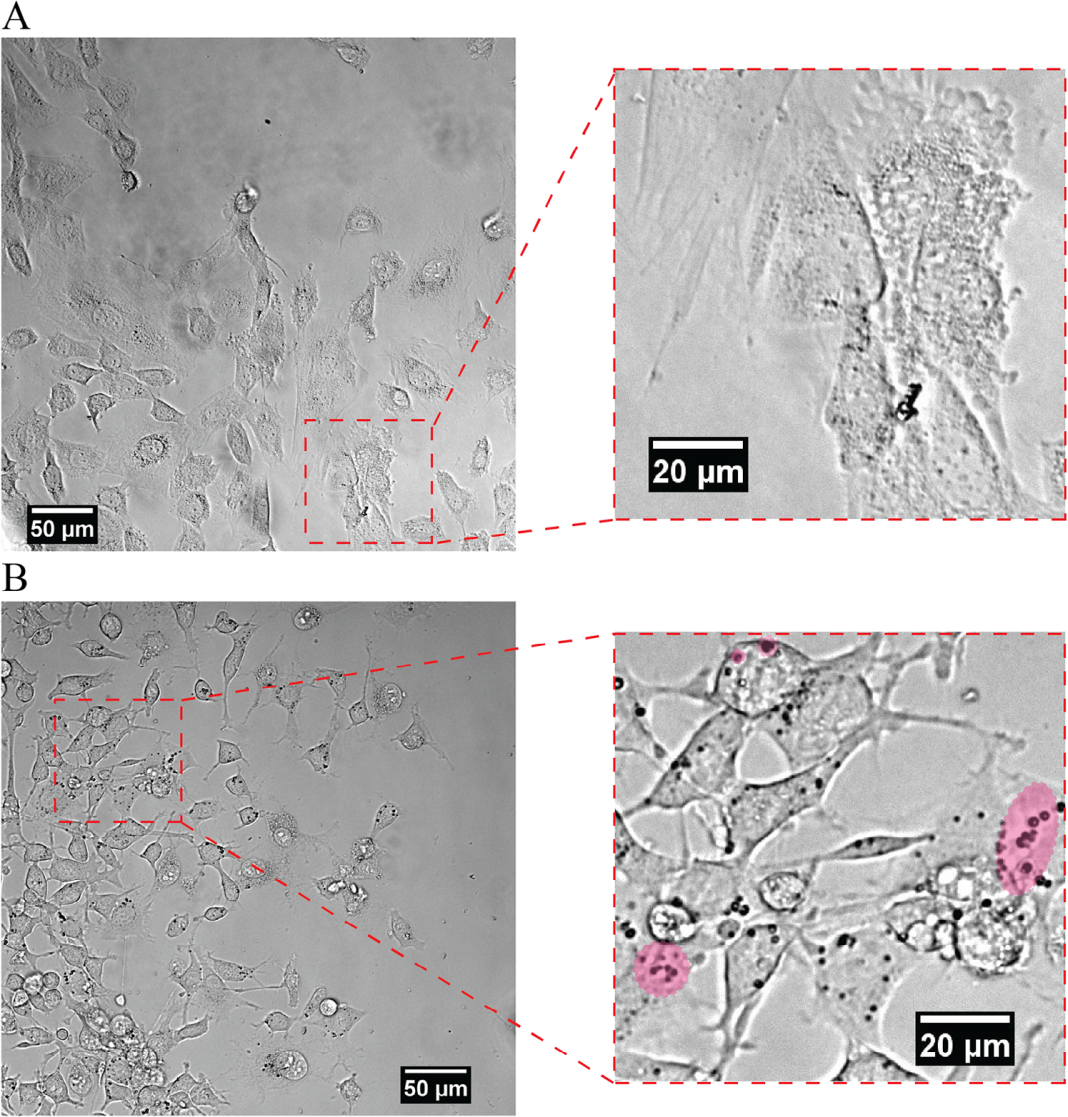
Bubble attachment to the cancer cells (in vitro assay). Bright‐field microscopy images of (A) healthy cells (NIH3T3) and (B) cancerous cells (CT26). While for the healthy cells, only a few bubbles are attached to the cells, there are significantly more bubbles attached to cancer cells. Inset images are 100 × 100 µm areas that highlight the attachment of the bubbles to the surface of the cancerous cells. Some groups of bubbles are highlighted with pink color.

Finding the desired target (biomarker) on the surface of the cells represents the most challenging part of developing a molecular imaging probe. The critical point in this process is the selective attachment of the moiety part on the surface of the microbubbles to the antigen on the surface of the cancer cells. To assess the selective attachment of our bubbles to a specific antigen, in vitro experiments were performed using a cancer cell line (CT26) having the targeted antigen and compared with a healthy cell line (NIH3T3) as a control. CT26 is a colon carcinoma cell line with biomarker CD87 on its surface.^[^ ^49^ ^]^ The CD87 cell surface receptor, also known as the Urokinase‐type plasminogen activator receptor (uPAR), is expressed at a high level in tumor cells, in contrast to healthy cells, where it is expressed at a low level.^[^ ^50^ ^]^ To target the CD87 antigen, the microbubbles (the shell adsorbed from a solution of 20% HFBI‐domZ + 80% HFBI) are decorated with IgG1k, an anti‐antigen monoclonal antibody. As explained in the experimental part, decorated bubbles were brought into contact with CT26 and NIH3T3 cells. **Figure** **5**A shows an optical microscopy image of a group of NIH3T3 cells (with CD87 antigens) after incubation with decorated bubbles and subsequent washing step. Barely any bubble attaches to the healthy NIH3T3 cells. In contrast, there are hundreds of bubbles attached to the cancerous CT26 cells (Figure 5B). This demonstrates the selective attachment of the decorated bubbles to the target cells. The inset images in Figure 5A,B cover a similar area (0.01 mm^2^), with a slight difference in cell coverage. The cell coverage is ≈60% in Figure 5A (NIH3T3 cells) and ≈70% in Figure 5B (CT26 cells). Despite the 10% difference in cell coverage, a clear disparity in the number of bubbles attached to the cells was observed. In the case of the CT26 cell line, ≈120 bubbles were attached to the cells within this area, whereas only three bubbles were attached in the same area for NIH3T3 cells. In addition, this may lead to the assumption of the programmability of our bubbles, which means that the bubbles could be decorated with any monoclonal anti‐antigen to target a specific surface receptor.

### 2.7 Bubble Echogenicity

As an ultrasound contrast agent, echogenic bubbles are an excellent tool for disease diagnosis. The physical properties of echogenic bubbles enable them to reflect ultrasound waves and thus appear as white area in the space where they are located.

**Figure 6:**
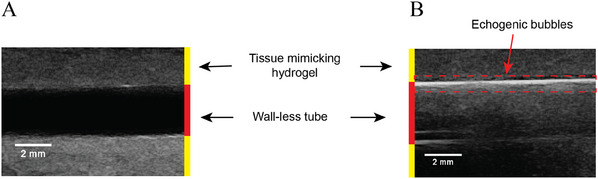
Echogenicity of bubbles. Ultrasound imaging of a wall‐less tube in a tissue‐mimicking hydrogel filled with PBS buffer solution (A) before and (B) after the injection of echogenic microbubbles coated with HFBI. After injection, a signal gradient is observed in the tube due to bubble reflection.

The echogenicity of HFBI stabilized bubbles was assessed using a portable ultrasound imaging device (Clarius Scanner L20 HD, Canada. Figure S5, Supporting Information). **Figure** **6** shows the bubbles in a wall‐less tube inside a tissue‐mimicking material consisting of an agar hydrogel containing graphite microparticles.^[^ ^51^ ^]^ The dark area in the left image corresponds to the tube filled only with PBS buffer solution before bubbles injection. After injection, a gradient of bubbles can be seen in the right image, with the highest concentration at the top of the tube (in white) and the lowest at the bottom (in black). Due to buoyancy, bubbles accumulate at the top, showing a clear difference between the tube and the tissue‐mimicking material (right side) compared to the bubble‐free image (left side).

## 3 Conclusion

Molecular diagnostic imaging based on echogenic microbubbles would represent an advance in disease diagnosis, allowing the detection of disease biomarkers at an early stage, which is crucial for disease control and treatment. This study focuses on the key features of a novel advanced ultrasound molecular imaging probe design as a non‐invasive and cost‐effective diagnostic modality. We demonstrated that specifically designed microbubbles could target the biomarker on the surface of murine colon cancer cells. The properties such as size, stability, echogenicity, and biomarker detection were investigated. To achieve high stability and good target accessibility, C_4_F_10_ gas bubbles encapsulated with a mixture of native‐type HFBI hydrophobin and HFBI‐domZ fusion protein were prepared, mainly having a size similar to that of red blood cells (between 7 and 10 µm). The stability of the bubbles under high pressure was tested using a custom‐made capillary system connected to an inverted optical microscope. The results confirmed that the bubbles survive under a pressure of 200 mbar, which is much higher than the typical human blood pressure (130 mbar). The bubbles were decorated with IgG antibody molecules, and the decoration was confirmed using confocal microscopy and cryo‐TEM. This suggests that bubbles can be redesigned to target different antigens. The echogenicity of the bubbles was tested using a portable ultrasound scanner to ensure their ability to serve as a signaling component for the ultrasound imaging molecular probe. Finally, the ability of the bubbles to serve as a moiety part for the probe was tested through in vitro experiments. Bubbles found the targeted antigen on the surface of cancer cells and bound to them, in contrast to healthy cells.

Based on these results, an advanced ultrasound imaging molecular imaging probe with programmability potential is developed and proposed for further in vivo experiments as an essential step before medical applications.

## 4 Experimental Section

### Materials

Perfluoro‐*n*‐butane 98% was purchased from ABCR (Karlsruhe, Germany). NHS‐Fluorescein antibody labeling kit was obtained from Thermo Fisher Scientific. IgG antibodies, POHG, and PBS were purchased from Sigma–Aldrich (Missouri, USA). Water was deionized using a Milli‐Q water purification system (Millipore) with a resistivity of ≈18.2 MΩ cm. Dulbecco's Modified Eagle's Medium (DMEM, Gibco), antibiotics (10000 µg mL^−1^ streptomycin and 100000 U mL^−1^ penicillin, Gibco), and fetal bovine serum (FBS, Gibco) were purchased from Fisher Scientific. Fibronectin was purchased from Sigma–Aldrich.

### Protein Expression and Purification

HFBI hydrophobin protein was produced and purified as described in Linder et al.^[^ ^52^ ^]^ In this work, a BL21star(DE3) (Invitrogen) E. coli strain carrying pMJS205 plasmid for codon‐optimized S. cerevisiae Erv1 + codon‐optimized human PDI^[^ ^53^ ^]^ was used to express HFBI‐DomZ protein from plasmid pHZ001 simultaneously. The plasmid pHZ001 was based on the pET28b+ expression vector. The expression vector carried a codon‐optimized version of the HFBI‐DomZ encoding gene cloned between NcoI and HindIII sites of pET28b+, thus adding a Hexa histidine tag in the C‐terminus of the HFBI‐DomZ fusion protein. The HFBI‐DomZ fusion consisted of *Trichoderma reesei* hydrophobin I (HFBI)^[^ ^42^ ^]^ and a Modified version of Staphylococcus aureus protein A B‐domain B, called Z‐domain (DomZ).^[^ ^44^ ^]^

For protein HFBI‐domZ expression, E. coli strain B7405 was cultured in a modified ZYM medium with 0.35% glucose at 37 °C at 220 rpm for the shake flask culture that was used as an inoculum.^[^ ^54^ ^]^ The final culture step was done in a 15‐liter volume in a stainless‐steel fermenter vessel (Braun Biostat) in ZYM modified with 0.05% glucose at 37 °C, pH 6.8. The cultivation time was 20 h.

The cells were simultaneously lysed and purified by aqueous two‐phase separation. First, the culture medium was supplemented with 137 mm NaCl, 10 mm Na_2_HPO_4_, 2.7 mm KCl, 1.8 mm H_2_PO_4_, 4% Triton X‐114, 10 mm PMSF, 10 mM EDTA and incubated overnight at room temperature under gentle shaking (80 rpm). To trigger the phase separation, the mixture was centrifuged for 20 min at 10000 g. Next, the lower phase was collected and washed with an equal volume of isobutanol to extract the hydrophobin fusion proteins and centrifuged again. Finally, the aqueous phase was collected and stored at −20 °C prior to further purification. The final purification step was done by immobilized metal affinity chromatography with a Fast Flow Chelating Sepharose column (GE) at pH 7.5 following the manufacturer's protocol. DG‐10 gel filtration columns (Bio‐Rad) were used to exchange the buffer with PBS (137 mM NaCl, 10 mm Na_2_HPO_4_, 2.7 mm KCl, 1.8 mm H_2_PO_4_). Finally, protein purity was assessed on SDS PAGE, and the concentration was determined with a spectrophotometer by measuring absorbance at 280 nm. The theoretical pI and Mw of HFBI‐domZ are 6.35 and 16 645.62 kDa, respectively. For the whole sequence of the HFBI‐domZ see Figure S6 (Supporting Information).

### Bubble Formation

1 ml of 0.2 mg  ml^−1^ solution consisted of 80% HFBI hydrophobin protein and 20% HFBI‐domZ fusion protein in PBS placed inside a 2.5 ml glass vial. The vial was sealed using an aluminum cap and a septum. The air inside the vail was replaced with C_4_F_10_ using a 50 ml Hamilton syringe. The vial was placed in a VialMix shaker and agitated for 45 s. A white suspension composed of nano and microbubbles was obtained.

### Surface Tension and Surface Dilatational Rheology

Static surface tension and mechanical properties of the HFBI‐domZ layer adsorbed at three non‐polar gases (air, C_4_F_10_, and SF_6_) interfaces were carried out using an optical tensiometer (Theta tensiometer, Biolin Scientific, Finland). The obtained data were analyzed using OneAttension software (Biolin Scientific). First, a hooked needle filled with one of these gases using a Hamilton syringe was immersed in a solution of HFBI‐domZ fusion protein (0.01 mg ml^−1^). Then, a 10 µl gas bubble was created at the tip of the needle. The bubble was monitored for ≈3 h to measure the static surface tension, employing the axisymmetric drop shape analysis. When the curvature of the surface tension reached a steady state, a sinusoidal volume disruption was applied using an integrated pulsated droplet module (PD‐200). The change in bubble volume was monitored for 40 s, and the loss and storage moduli were collected using the same software (One Attention, Figure 1A–C).

### Bubble Size Distribution

Bubble size distribution was measured by laser diffraction using the Mastersizer 2000 (Malvern Panalytical, UK). Each sample was measured at least thrice, with three runs in each measurement. The stirrer speed was 2000 rpm. Refractive indexes of 1 and 1.33 were used for the dispersed phase (air) and dispersant (water), respectively. A volume‐based distribution was obtained.

### Zeta Potential

The bubbles’ ZP was measured using a Malvern Zetasizer Nano ZS90 (Malvern Panalytical, UK) based on the electrophoretic mobility principle. Samples were measured in a micro cuvette using a dip cell with an equilibration time of 2 min at 25 °C. Three runs of at least 30 continuous readings were obtained for each sample. Smoluchowski equation was used for ZP calculation.

### Confocal Microscopy

Three batches of protein solutions consisting of 100% native‐type HFBI, 80% HFBI + 20% HFBI‐domZ, and 50% HFBI + 50% HFBI‐domZ were prepared to create microbubbles (as explained in the formation of bubbles section). The antibody molecules labeling procedure was executed according to the protocol in the labeling kit (supplementary data). Bubble solutions were diluted in 40 ml of distilled water and centrifuged for 1 min at 48 RCF. Then, 5 ml from the top of the supernatant was mixed with 10 µl of IgG‐NHS‐Fluorescein (2 mg ml^−1^), and the mixture was incubated for 20 min at room temperature, protected from light. After that, the mixture was diluted in 40 ml of distilled water and centrifuged for 1 min at 48 RCF. The supernatant of the solution was collected and used for the confocal microscopy experiments. The samples observed by confocal microscopy were capillaries filled by the capillary effect by immersing them in the top of the supernatant.

Bubbles in the capillaries have been observed by confocal microscopy (LSM 710 from Zeiss, Germany). Experiments were performed with *λ_ex_* = 488 nm and *λ_em_* = 490 – 753 nm. The samples were observed the same day and the same parameters were used for each sample to compare signal intensities.

### Atomic Force Microscopy

A 100 µl droplet made of a mixture of HFBI native‐type protein (0.2 mg ml^−1^) and HFBI‐domZ (0.2 mg ml^−1^) fusion protein mixtures was placed on a parafilm substrate for ≈1 h to get a flattened area on top of the droplets and to ensure that a monolayer was presented at the interface. Then, a highly ordered pyrolytic graphite (HOPG, ZYA quality) substrate was brought into contact with the top of the droplets. The substrate with a hydrophobin monolayer was gently washed with 200 ml of distilled water. The sample was placed on the AFM stage for ≈30 min before use to ensure that the sample was dehydrated.

AFM measurements were carried out using a Dimension Icon AFM (Bruker AXS, France; formerly Veeco) with a ScanAsyst‐air cantilever (sharp silicon nitride tip with a nominal radius of 2 nm for PeakForce Tapping in the air). The scan size was set to 100 nm × 100 nm with a 256 × 256 pixels resolution, and the scan was done at a frequency of 1 kHz. The ScanAsyst Auto control was set to “individual” with a PeakForce Amplitude of 170 nm. The spring constant and PeakForce frequencies were 0.4 N m^−1^ and 2 kHz for all samples. Individual scans were performed for each sample at multiple locations on the surface.

### Cryo‐TEM

5 µl droplets of nanobubble dispersions were deposited on a lacey carbon treated with plasma (Electron Microscopy Sciences). Droplets were blotted with filter paper, plunged into liquid ethane (−170 °C) using an automatic plunger freezer (EM GP2, Leica), and stored in liquid nitrogen. The samples were visualized using a JEM‐3200FSC (JEOL) microscope at 300 kV acceleration voltage, and images were acquired using Digital Micrograph software (Gatan).

### Pressure Experiments

Microfluidic devices were prepared by UV‐curing a rectangular glass capillary (CM Scientific, 0.2 × 2.0 × 50 mm) on a microscopy slide and a needle (25G × 5/8″) on one side of the capillary. The needle was then connected to a reservoir via PTFE tubing (ID ≈250 µm). The reservoir was filled with PBS and connected to a piezoelectric pressure controller (Elveflow OB1 MK3+). The tubing and the capillary were filled with PBS, then a droplet of bubbles was deposited on the other side of the capillary (the side not connected to the tubing) and carefully aspired in the capillary. After the bubbles had been aspirated, the capillary was sealed using a thermosensitive wax (PELCO Quickstick 135).

The desired pressures were applied using the pressure controller software (ESI). Experiments were observed with an inverted microscope (Nikon Eclipse Ti), and bright‐field images of the bubbles were recorded with an sCMOS camera (Andor Zyla 4.2) operated using µManager (2.0 beta) open‐source microscopy software. Images were exported and analyzed using ImageJ.

### Cell Experiments

Noncancerous murine fibroblast NIH3T3 cells and mouse colon carcinoma CT26 cells (ATCC CRL‐2638; American Tissue Culture Collection) were used in the study. Cells were cultured at 37 °C under a 95% air/5% CO_2_ atmosphere in a culture medium consisting of DMEM supplemented with 10% (v/v) FBS and antibiotics (100 µg mL^−1^ streptomycin and 100 U mL^−1^ penicillin).

Observation chambers were prepared as follows. Circular 25‐mm cover glasses were sonicated in ethanol for 5 min, dried at room temperature, and plasma‐cleaned (Henniker plasma HPT‐100) for 10 min. The cover glasses were coated with a 0.1 mg l^−1^ solution of fibronectin (Sigma–Aldrich) in PBS for 1 h (Figure S7, Supporting Information).

The observation chambers were filled with DMEM containing either NIH3T3 cells (with a concentration of 10^7^ cells ml^−1^) or CT26 cells (with a concentration of 5 × 10^6^ cells ml^−1^). After 1 day of incubation at 37 °C with 5% CO_2_, the medium was removed from the chambers, the chambers were washed with PBS, and then filled with a bubble solution. The chambers were then closed with a 30‐mm cover glass on the upper side, and the magnetic chambers were flipped upside down so that the cells were in contact with the bubbles (Figure S5, Supporting Information). The chambers were then placed for 30 min in the incubator, after which the observation chambers were flipped again so that the cells were at the bottom of the observation chambers. Finally, the supernatant was discarded to remove the unattached bubbles, the surface of the observation chamber was washed using a PBS solution, and the cells were observed by bright field microscopy.

### Tissue Mimicking Material

Graphite agar phantom was prepared as follows. 3 g of agar were mixed with 150 ml of degassed distilled water. The mixture was heated to 75 °C on a hot plate with a magnetic stirrer. After that, 4.3 g of graphite was added to the mixture and heated to 95 °C. The mixture was then poured into molds and stored in a cool water bath.^[^ ^51^ ^]^

### The Echogenicity of the Bubbles

The graphite agar phantom that has a tube‐like empty space in the middle with a diameter ≈3 mm was placed on a plastic Petri dish. A portable ultrasound imaging device (Clarius Scanner L20 HD, Canada) was maintained in contact with the phantom from the top as shown in Figure S5 (Supporting Information). To avoid any empty space between the phantom and the device, some of ultrasound transmission gel was placed in between. After that, a suspension of HFBI‐coated bubbles, prepared as described in the bubble formation section, was drawn into a syringe and injected inside the phantom, and imaged. As a control experiment, a buffer solution (PBS) was injected without any bubbles.

## Conflict of Interest

The authors declare no conflict of interest.

## Supporting information



Supporting Information

## Data Availability

The data that support the findings of this study are available from the corresponding author upon reasonable request.
