# Supplementary material for: Hydrophobin‐Coated Echogenic Microbubbles for Molecular Targeting of Tumor Cells
Source: Adv Sci (Weinh). 2025 May 19;12(22):2401526. doi: 10.1002/advs.202401526 (PMC12165063; doi:10.1002/advs.202401526)
Supplement: Supplementary file 1 — Supporting Information [file ADVS-12-2401526-s001.pdf]

ADVANCED SCIENCE
Open Access

Supporting Information

for *Adv. Sci.*, DOI 10.1002/advs.202401526

Hydrophobin-Coated Echogenic Microbubbles for Molecular Targeting of Tumor Cells

*Hedar H. Al-Terke, Grégory Beaune, Muhammad Junaid, Jani Seitsonen, Arja Paananen, Pierangelo Metrangolo, Jaakko V. I. Timonen, Jussi Joensuu and Robin H. A. Ras**

Supporting Information

Hydrophobin-coated echogenic microbubbles for molecular targeting of tumor cells

Hedar H. Al-Terke,[1,2] *Grégory Beaune,*[1,2] *Muhammad Junaid,*[1,2] *Jani Seitsonen,*[1] *Arja Paananen,*[2,3] *Pierangelo Metrangolo,*[4] *Jaakko V. I. Timonen,*[1,2] *Jussi Joensuu,*[3] *Robin H. A. Ras.*[1,2]

1 Department of Applied Physics, Aalto University School of Science, FI-02150, Espoo, Finland.
2 Center of Excellence in Life-Inspired Hybrid Materials (LIBER), FI-02150, Espoo, Finland.
3 VTT Technical Research Centre of Finland Ltd. P.O.Box 1000, FI-02044 VTT, Espoo, Finland.
4 Department of Chemistry, Materials, and Chemical Engineering "Giulio Natta," Politecnico di Milano, IT-20131, Milano, Italy.
Email. robin.ras@aalto.fi

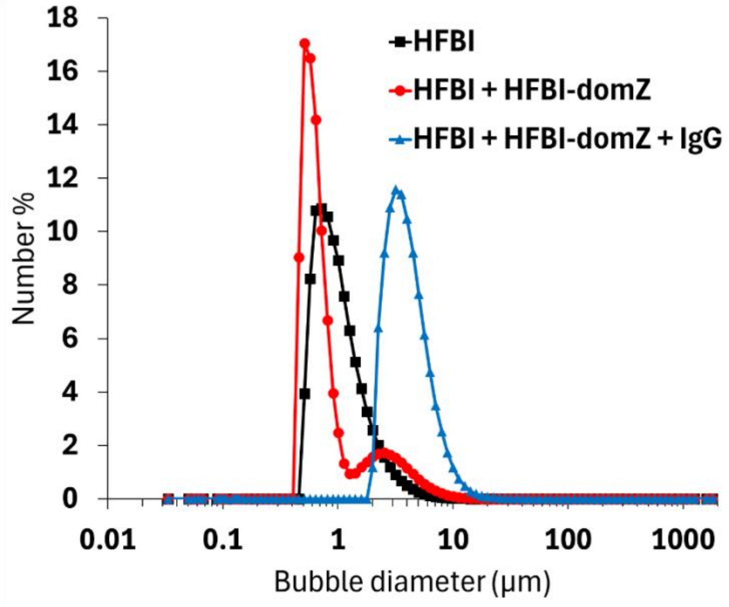

Figure S1. Bubble size distribution measured using a Mastersizer particle analyzer for HFBI (in black), HFBI + HFBI-domZ (in red), and HFBI + HFBI-domZ + IgG (in blue) formulations.

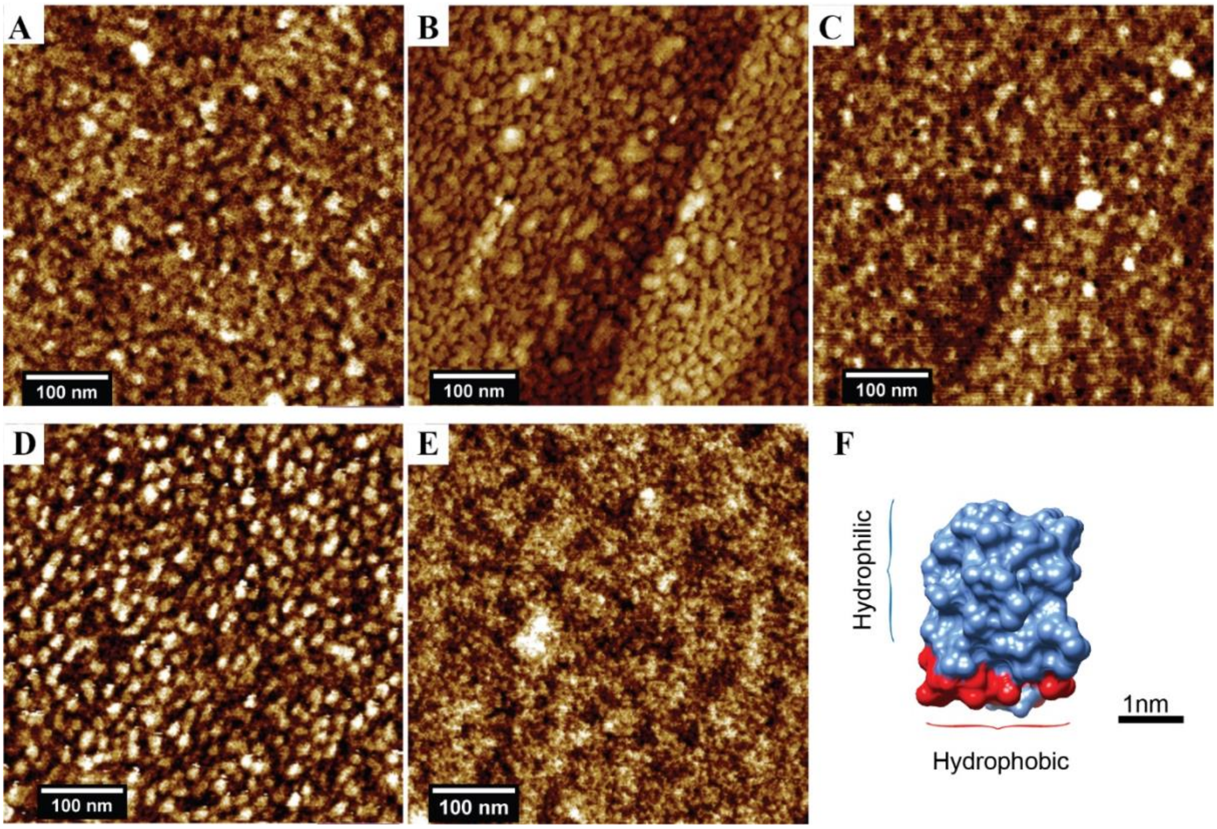

Figure S2. Atomic force microscopy (AFM) images of native-type hydrophobin (HFBI) and fusion protein (HFBI-domZ) with different ratios. (A) 20% HFBI-domZ and 80% HFBI. (B) 40% HFBI-domZ and 60% HFBI. (C) 60% HFBI-domZ and 40% HFBI. (D) 80% HFBI-domZ and 20% HFBI. (E) 100% HFBI-domZ. All the samples were formed on a highly oriented pyrolytic graphite (HOPG) substrate. (F) The 3D structure of a single molecule of HFBI protein (PDB ID 2FZ6). The 3D image was created using Chimera software.

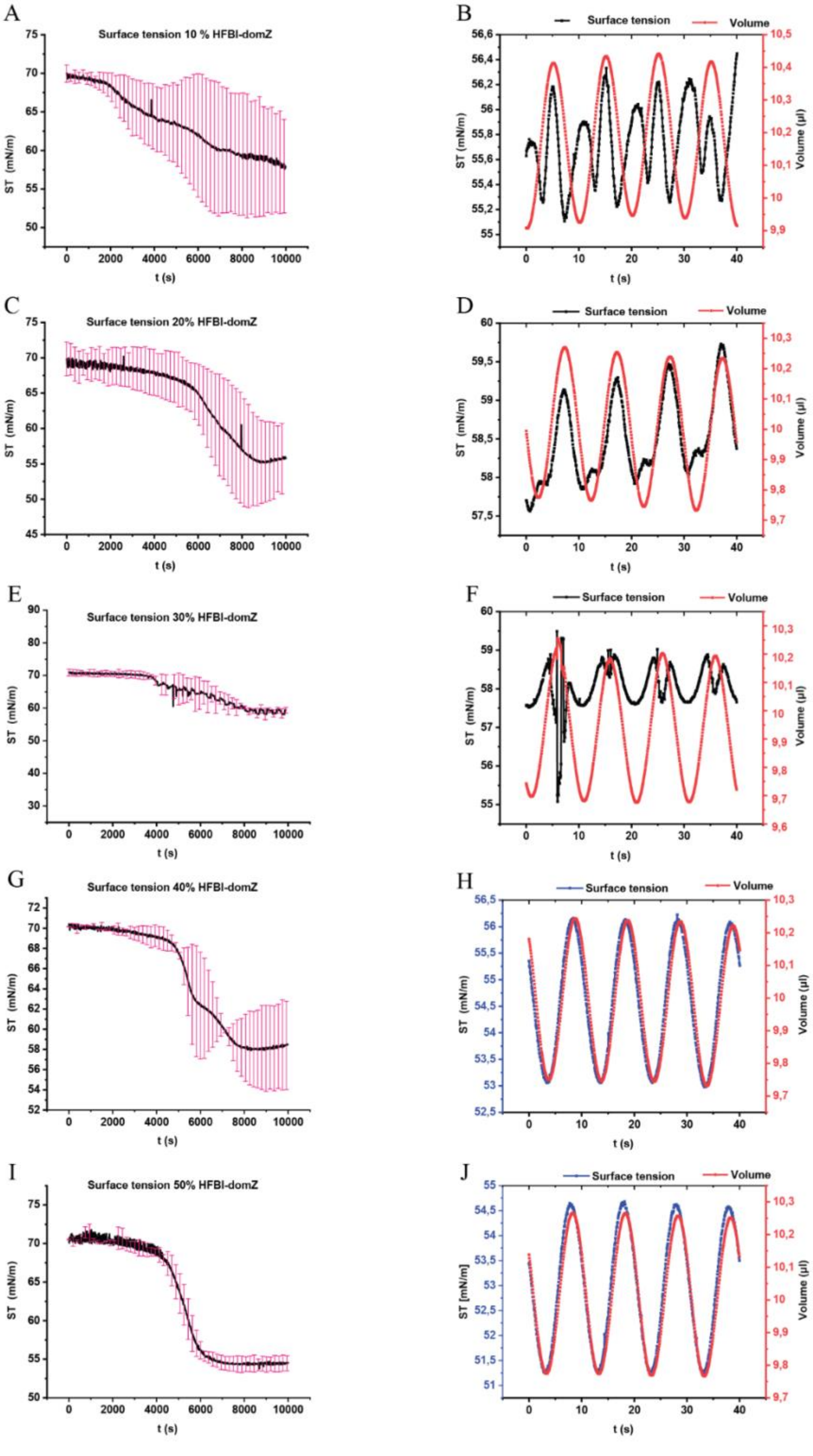

Figure S3. Surface tension (ST) and surface dilatational rheology of HFBI and HFBI-domZ mixture solutions with five different ratios. A, C, E, G, and I show the surface tension of HFBI and HFBI-domZ mixture with varying ratios of percentage, 10, 20, 30, 40, and 50 percent of HFBI-domZ in the solution, respectively. B, D, F, H, and J show the surface dilatational rheology measurements. In G and I, the surface tension curves show the regular shape of the surface tension carve (smooth three phases curve) for surface active materials, meaning that the bubbles shape fit well the Laplace equation model in the software. At the same time, H and J show a good match between the values of surface tension (blue curve) and the volume. Only these two ratios allowed us to measure the viscoelastic properties of the layers at the interface.

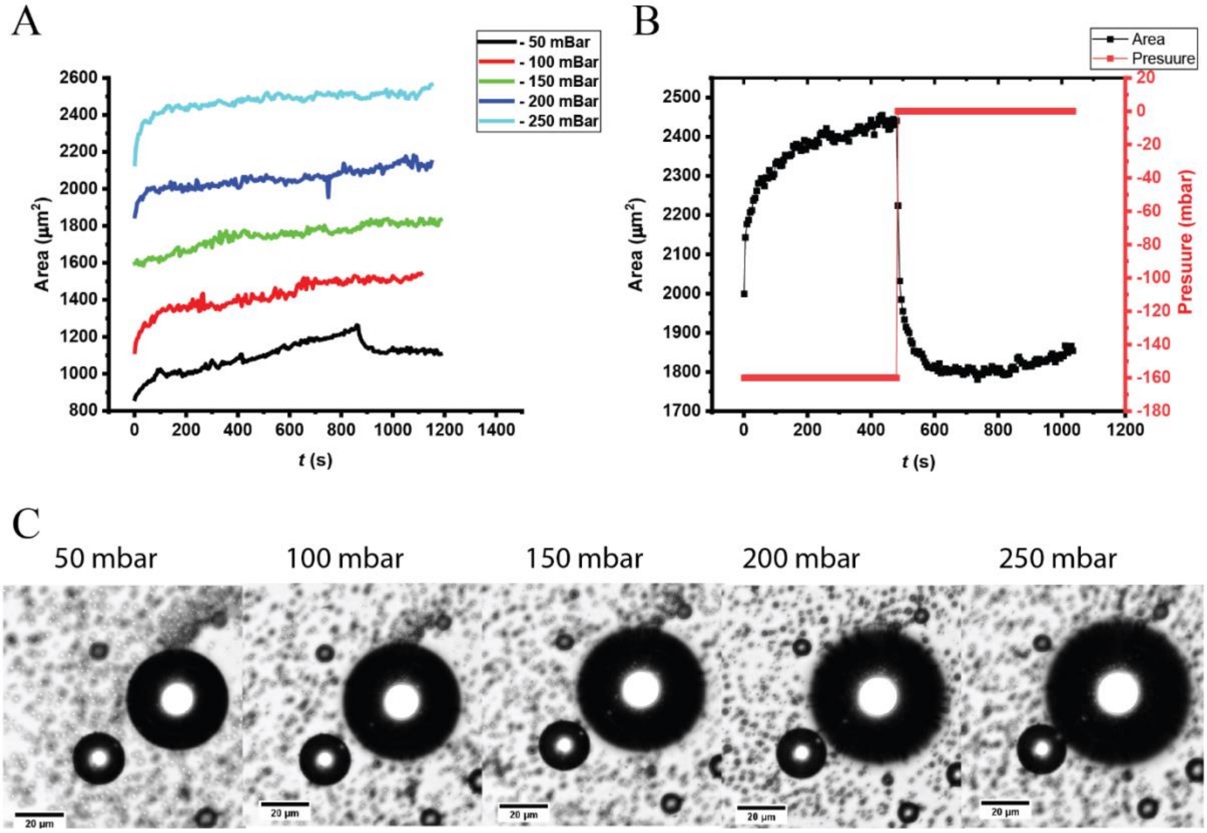

Figure S4. Aspiration pressure experiments. (A) the cross-section area of the bubble increases as the aspiration pressure ΔP_{asp} inside the capillary increases in 50 mbar steps. (B) the cross-section area of the bubble at ΔP_{asp} = 160 and 0 mbar. Finally, (C) the final size of the bubbles for different aspiration pressures between 50 and 250 mbar.

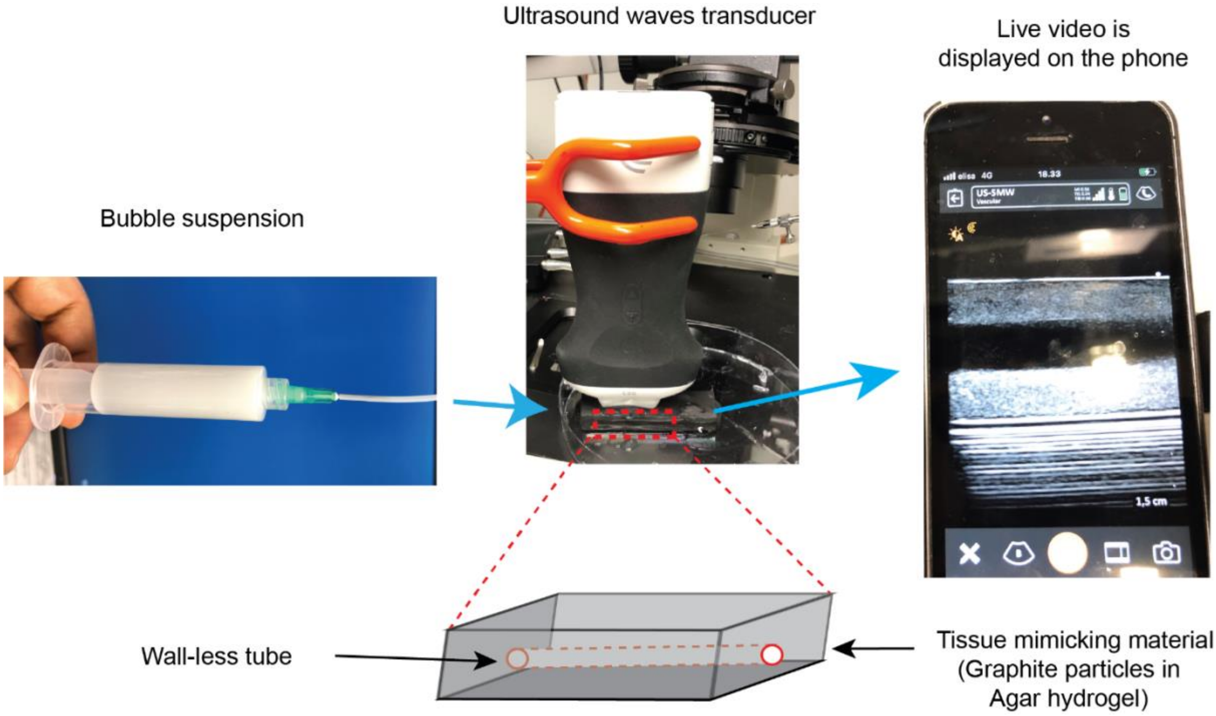

Figure S5. Experimental setup for ultrasound imaging.

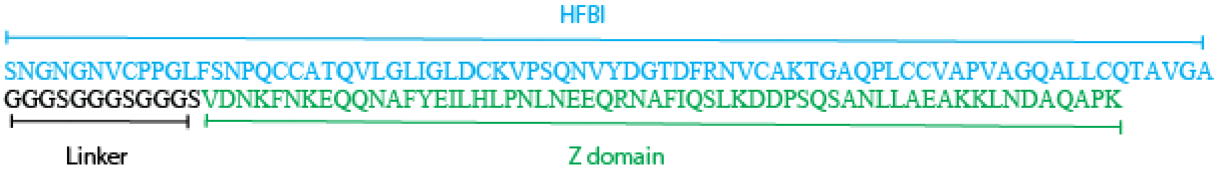

Figure S6. Sequence of the HFBI-domZ fusion protein**.**

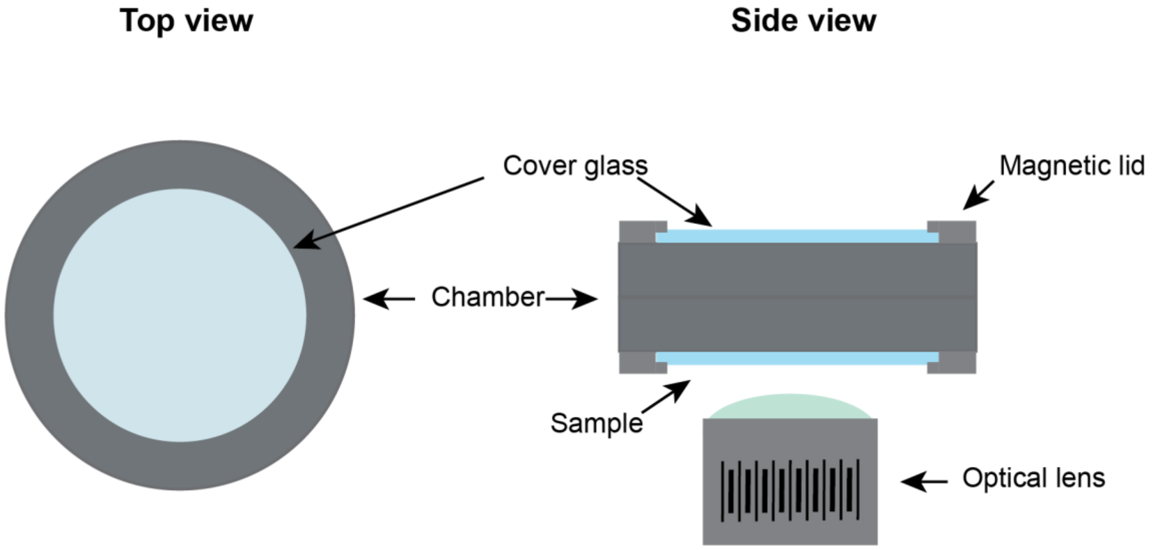

Figure S7. Experimental setup for in-vitro imaging.

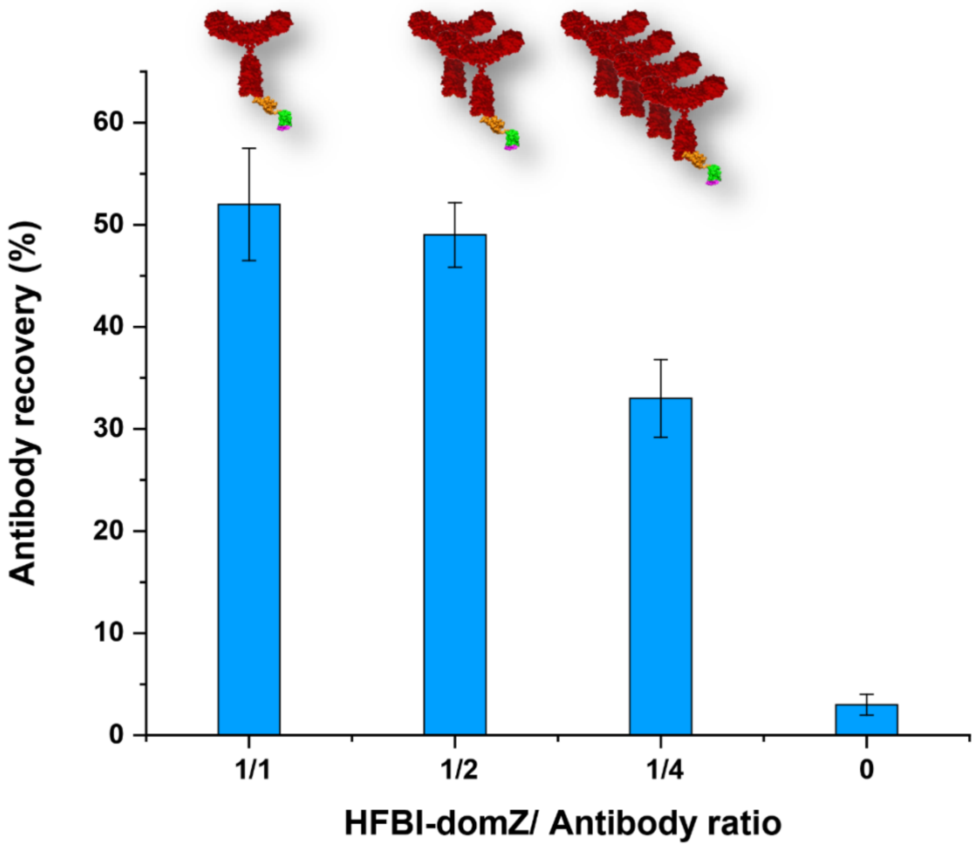

Figure S8. Monoclonal antibody molecules recovery using four different HFBI-domZ ratios (1:1, 1:2, 1:4, and 0). The columns represent mean ($n = 3$), and the error bars stand for standard deviation.

Table S1. Suitable bubble size percentage of bubbles for different medicine related applications.

Application [a]	Formulation	Size range [a]	Bubbles (volume)	Bubbles (number)
Extravascular transport	HFBI	≤ 0.5 μm	0 %	0 %
Cancer theragnostic	HFBI	≤ 1 μm	4 %	63 %
Blood perfusion<br>Inflammation<br>Tumor angiogenesis	HFBI	≤ 10 μm	74 %	99.9 %
Ultrasound imaging	HFBI	1 – 10 μm	70 %	40 %
	HFBI + HFBI-domZ + IgG	1 – 10 μm	44 %	98 %

[a] Applications and corresponding sizes according are from [48].
[b] Determined using the Mastersizer particle analyzer (Figure 2B, S1).

1. Antibody molecules labeling

40 µl of borate buffer (0.67 M) were added to 500 µl of IgG molecules in PBS solution (2 mg ml^{-1}, pH = 7.3). The mixture was added to the reagent vial (provided by the supplier, Thermo Fisher Scientific), mixed with a pipet, and vortexed for a few seconds. The vial was briefly centrifuged and the sample at the bottom was collected. The sample was then incubated at room temperature and protected from light for 60 min. Two spin columns were filled with 400 µl of purification resin and centrifuged at 1000 g for 30 s. Finally, the 540 µl of IgG and reagent solution were divided into two parts of equal volume and centrifuged for 30 s at 1000 g, yielding approximately 500 µl of labeled IgG molecules.

2. Self-organization of protein molecules

Proteins and their variants usually tend to self-organize at interfaces. The materials used to create the bubbles are a mixture of the native hydrophobin protein HFBI and the fusion protein HFBI-domZ. Both are highly surface active, even though the molecular size of HFBI-domZ is almost double that of the HFBI molecule (about 14 kDa and 7.4 kDa, respectively). Therefore, it was necessary to confirm the existence of both proteins at the interface. Atomic force microscopy is used to visualize the self-organized layers of the HFBI and HFBI-domZ mixtures at the air-water interface to see the molecular structure of the layer (see Experimental section). The images in Figure S2 from A to E represent the layer structure of the HFBI-domZ and HFBI mixtures. The mixing percentages were 20%, 40%, 60%, 80%, and 100% HFBI-domZ. All of these images show two main regions: the light region and the dark region, which means that there are two different sizes of molecules, which can prove the existence of both proteins at the interface. The dark areas are assumed to be sample preparation defects.

3. Antibody capture by two-phase extraction

The HFBI–domZ fusion protein can bind to and capture monoclonal antibodies (mAb) from hybridoma culture supernatant in aqueous two-phase system (ATPS). Different HFBI-domZ : mAb ratios were tested. It was found that roughly half of the mAb can be recovered in ATPS system with molecular ratios up to 1/2 (mAb/HFBI-domZ). Reduction of HFBI-DomZ amount to 1/4 reduces mAb capture to 33%. While merely 3% of the antibody amount was detected in the absence of HFBI-DomZ (Figure S8).

To capture antibodies from the hybridoma culture supernatant, a molar ratio of 1:1, 1:2 and 1:4 between HFBI–domZ and mAb was used. In detail, solution of purified HFBI–domZ was added to 0.4 ml of concentrated hybridoma culture supernatant containing 4.7 mg/ml of anti-Chlamydia 6709 mAb (Medix Biochemica) and topped to 1 ml with PBS buffer. Prior to the addition of 6% (w/v) Triton X-114, the mixture was warmed to 24 °C for 5 min. After mixing 15 min at 24 °C in a rotary drum, the phases were separated by centrifugation at 4000 g for 5 min. The aqueous phase was removed, and the surfactant phase was supplemented with equal volume of 0.05 M glycine–HCl, pH 2.2 to release antibodies from HFBI–Protein A (final pH at this point 3.0). The mixture was incubated in the rotary drum at 24 °C for 5 min and centrifuged as above. The aqueous phase containing the purified antibodies was collected and neutralized to pH 8 with 18 µl of 1M Tris–HCl pH 8.5. Fractions of samples before and after the purification procedure were analysed by SDS–PAGE, scanned and mAb amount quantified by densitometry (Totallab software). All samples were analyzed as technical triplicates.
